# Endoscopic submucosal dissection of an atypical rectal submucosal lesion: a rare case of a large digestive angiodyplasia of exclusive submucosal presentation

**DOI:** 10.1055/a-2055-9846

**Published:** 2023-03-30

**Authors:** Jean Grimaldi, Louis-Jean Masgnaux, Timothée Wallenhorst, Valérie Hervieu, Jérémie Jacques, Jérôme Rivory, Mathieu Pioche

**Affiliations:** 1 Endoscopy and Gastroenterology Unit, Edouard Herriot Hospital, Hospices Civils de Lyon, Lyon, France; 2 Gastroenterology and Endoscopy Unit, Pontchaillou University Hospital, Rennes, France; 3 Institute of Multi-Site Pathology of the HCL-Est Site, GHE University Hospital, Bron, France; 4 Gastroenterology and Endoscopy Unit, Dupuytren University Hospital, Limoges, France

We report here the case of a 37-year-old patient without comorbidities referred to our unit for endoscopic submucosal dissection (ESD) resection of a submucosal lesion of the lower rectum. The lesion measured 40 × 30 mm and did not present any mucosal abnormality ( Fig. 1 , Fig. 2 ). An echo-endoscopy was performed prior to resection, showing an atypical anechogenic duct within the lesion ( Fig. 3 ) without clear blood flow on Doppler analysis, which initially led to the suspicion of an ectopic gland or a fistulated rectal gland.

**Fig. 1:**
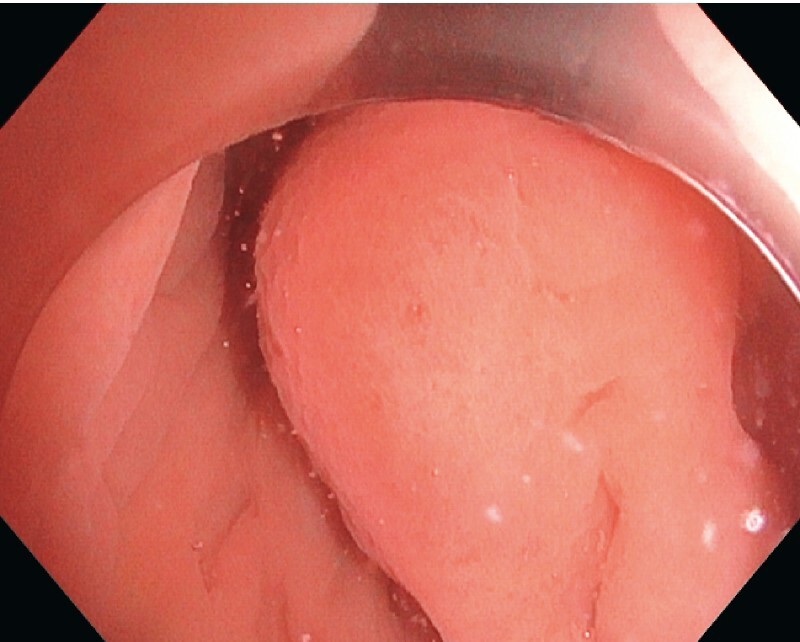
Endoscopic view of the lesion in white light.

**Fig. 2:**
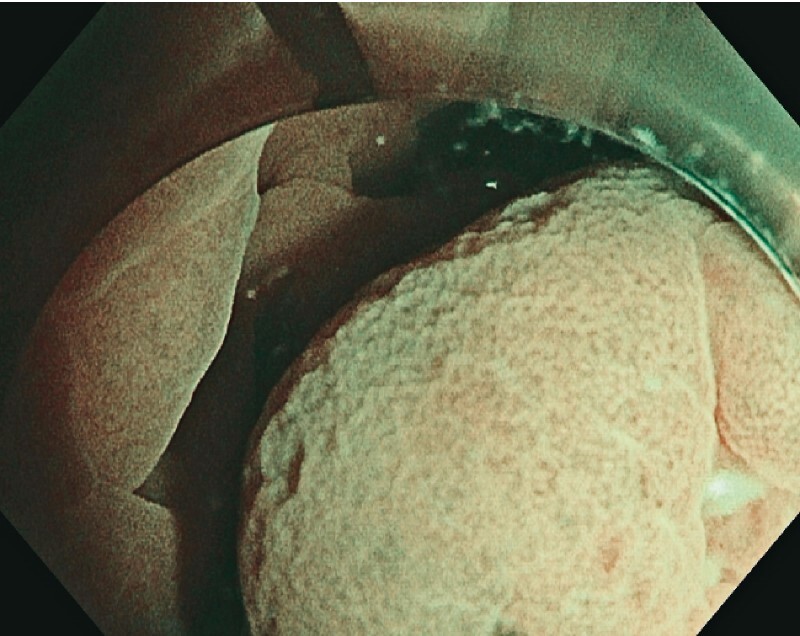
Endoscopic view of the lesion in narrow-band imaging, showing no abnormalities of the pit or vascular pattern.

**Fig. 3:**
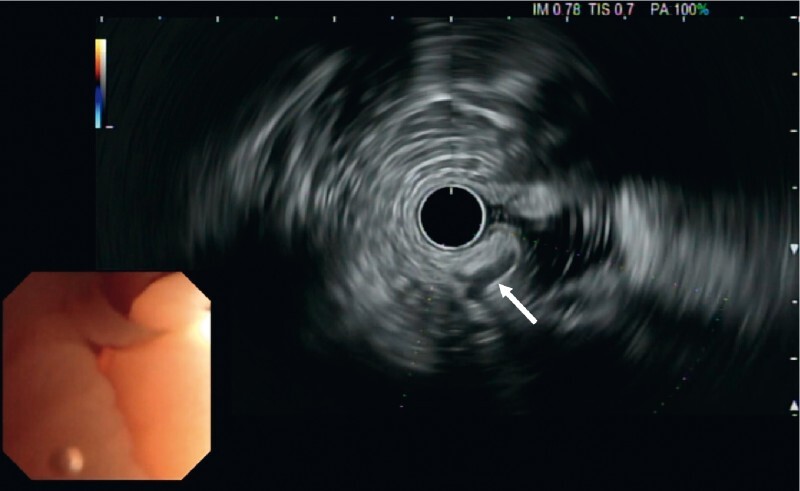
Echo-endoscopic view of the lesion showing an atypical anechogenic duct within the lesion, which initially led to the suspicion of an ectopic gland or a fistulated rectal gland.

We then decided to perform ESD. During the procedure, a large vessel was identified in the center of the lesion and was coagulated with the knife ( Video 1 ). The entire dissection took 20 minutes. There were no complications after the procedure, including hemorrhage.

**Video 1** Endoscopic submucosal dissection of an atypical rectal submucosal lesion: a rare case of a large digestive angiodyplasia of exclusive submucosal presentation.


Histology revealed an intestinal angiodysplasia of submucosal development ( Fig. 4 ) with complete resection. To our knowledge, this is the first description of an intestinal angiodysplasia presenting as a submucosal tumor.

**Fig. 4:**
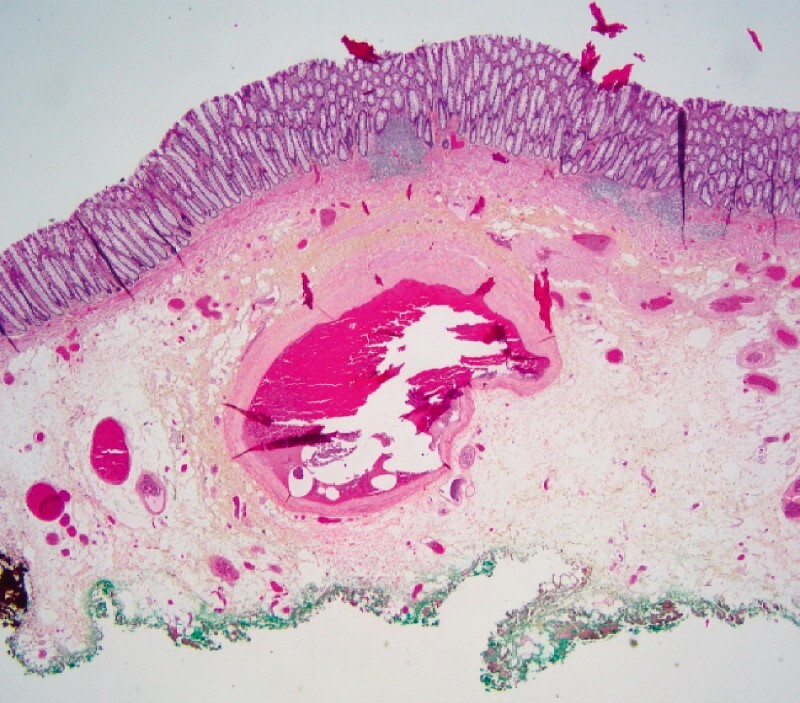
Histologic view of the lesion (stained with hematoxylin phloxine and safranin). Angiodysplasia consists of dilated, distorted ,and tortuous vessels lined by non-atypical endothelium and, here, thick small muscle walls located in the submucosae. Here, the mucosae are normal without inflammation or vessel abnormalities.

The conventional treatment for mucosal intestinal angiodysplasia is argon plasma coagulation. This treatment is not without morbidity and does pose significant risks of perforation and delayed bleeding, especially for large lesions 1 . The treatment by mucosectomy of colonic angiodysplasias has already been described in case treatment by argon plasma coagulation fails 2 . In the case of large angiodysplasias or in this specific case of a pure submucosal lesion, ESD could offer the possibility of targeting coagulation on the feeding vessel 3 .

Further studies are needed to evaluate the efficacy in terms of recurrence rate of the submucosal dissection technique as well as its safety in the treatment of large intestinal angiodysplasias.

Endoscopy_UCTN_Code_TTT_1AQ_2AD

## Notes

Endoscopy E-Videos

https://eref.thieme.de/e-videos
*Endoscopy E-Videos* is an open access online section, reporting on interesting cases and new techniques in gastroenterological endoscopy. All papers include a high quality video and all contributions are freely accessible online. Processing charges apply, discounts and wavers acc. to HINARI are available. This section has its own submission website at https://mc.manuscriptcentral.com/e-videos
